# Comparative assessment of absolute cardiovascular disease risk characterization from non-laboratory-based risk assessment in South African populations

**DOI:** 10.1186/1741-7015-11-170

**Published:** 2013-07-24

**Authors:** Thomas A Gaziano, Ankur Pandya, Krisela Steyn, Naomi Levitt, Willie Mollentze, Gina Joubert, Corinna M Walsh, Ayesha A Motala, Annamarie Kruger, Aletta E Schutte, Datshana P Naidoo, Dorcas R Prakaschandra, Ria Laubscher

**Affiliations:** 1 Divisions of Cardiovascular Medicine, Brigham and Women’s Hospital, 75 Francis Street, Boston, MA 02115, USA; 2 Department of Public Health, Division of Health Policy, Weill Cornell Medical College, 402 E. 67th Street, New York, NY 10065, USA; 3 Department of Medicine, University of Cape Town, Observatory, Cape Town 7925, South Africa; 4 Department of Internal Medicine, School of Medicine, University of the Free State, 205 Nelson Mandela Drive, Park West, Bloemfontein 9301, South Africa; 5 Department of Biostatistics, University of the Free State, 205 Nelson Mandela Drive, Park West, Bloemfontein 9301, South Africa; 6 School of Tourism, Hospitality and Sport, Technikon Free State, 20 President Boshof Street, Bloemfontein 9320, South Africa; 7 Department of Endocrinology, Nelson R. Mandela School of Medicine, University of KwaZulu-Natal, Mazisi Kunene Road, Durban 4041, South Africa; 8 Faculty of Health Sciences-Africa Unit for Transdisciplinary Health Research, North-West University, Hofman Street, Potchefstroom 2531, South Africa; 9 Hypertension in Africa Research Team, North-West University, Potchefstroom 2531, South Africa; 10 Department of Cardiology, University of KwaZulu-Natal, Inkosi Albert Luthuli Central Hospital, Bellair Road, Durban 4041, South Africa; 11 Department of Biomedical and Clinical Technology, Durban University of Technology, University of KwaZulu-Natal, Mazisi Kunene Road, Durban 4041, South Africa; 12 Biostatistics Unit, Medical Research Council, Francie van Zijl Drive, Tygerberg 7505, South Africa; 13 Chronic Diseases Initiative for Africa, Department of Medicine, University of Cape Town, Observatory, Cape Town 7925, South Africa

**Keywords:** Cardiovascular disease, Prevention, Cholesterol, Stroke, Coronary heart disease

## Abstract

**Background:**

All rigorous primary cardiovascular disease (CVD) prevention guidelines recommend absolute CVD risk scores to identify high- and low-risk patients, but laboratory testing can be impractical in low- and middle-income countries. The purpose of this study was to compare the ranking performance of a simple, non-laboratory-based risk score to laboratory-based scores in various South African populations.

**Methods:**

We calculated and compared 10-year CVD (or coronary heart disease (CHD)) risk for 14,772 adults from thirteen cross-sectional South African populations (data collected from 1987 to 2009). Risk characterization performance for the non-laboratory-based score was assessed by comparing rankings of risk with six laboratory-based scores (three versions of Framingham risk, SCORE for high- and low-risk countries, and CUORE) using Spearman rank correlation and percent of population equivalently characterized as ‘high’ or ‘low’ risk. Total 10-year non-laboratory-based risk of CVD death was also calculated for a representative cross-section from the 1998 South African Demographic Health Survey (DHS, n = 9,379) to estimate the national burden of CVD mortality risk.

**Results:**

Spearman correlation coefficients for the non-laboratory-based score with the laboratory-based scores ranged from 0.88 to 0.986. Using conventional thresholds for CVD risk (10% to 20% 10-year CVD risk), 90% to 92% of men and 94% to 97% of women were equivalently characterized as ‘high’ or ‘low’ risk using the non-laboratory-based and Framingham (2008) CVD risk score. These results were robust across the six risk scores evaluated and the thirteen cross-sectional datasets, with few exceptions (lower agreement between the non-laboratory-based and Framingham (1991) CHD risk scores). Approximately 18% of adults in the DHS population were characterized as ‘high CVD risk’ (10-year CVD death risk >20%) using the non-laboratory-based score.

**Conclusions:**

We found a high level of correlation between a simple, non-laboratory-based CVD risk score and commonly-used laboratory-based risk scores. The burden of CVD mortality risk was high for men and women in South Africa. The policy and clinical implications are that fast, low-cost screening tools can lead to similar risk assessment results compared to time- and resource-intensive approaches. Until setting-specific cohort studies can derive and validate country-specific risk scores, non-laboratory-based CVD risk assessment could be an effective and efficient primary CVD screening approach in South Africa.

## Background

The multiple risk factors for cardiovascular diseases (CVD) frequently occur simultaneously in individuals at risk. Because of this clustering, risk factors interact synergistically to increase the level of CVD risk to such an extent that the resulting absolute risk levels achieved are much higher than would be anticipated when considering the individual risk factors. These patients with the highest level of absolute CVD risk will benefit substantially if they are identified and all their risk factors diagnosed early and treated appropriately [1]. In fact, such an absolute CVD risk approach has been shown to be more cost-effective than treating patients’ individual risk factors to prevent CVD [2]. The calculation of the absolute CVD risk is usually based on age, gender, tobacco use status, blood pressure levels and blood cholesterol levels as was done with data from the Framingham study [3]. A number of similar formulations of absolute CVD risk have been proposed in a variety of settings where cohort studies were conducted to evaluate the usefulness of absolute CVD risk scores in predicting CVD events [4-7].

The absolute risk determination approach holds particular promise for resource scarce settings where the limited resources could best be utilized by identifying those at highest absolute CVD risk. However, blood lipid determinations for screening purposes will be far too costly in most developing country settings with limited resources and consequently are unlikely to be adopted as policy in these settings. This has prompted an investigation into the possibility of using other known CVD risk factors that are easier and less costly to measure in the place of CVD risk factors that require costly laboratory tests when calculating absolute CVD risk scores. This work compared the ability to predict first time fatal and non-fatal CVD events in the National Health and Nutrition Examination Survey (NHANES) I follow-up study cohort by two risk prediction models: the laboratory-based Framingham risk score and a non-laboratory-based model, which required only history and physical examination measures [8]. The non-laboratory-based model used the same risk factors but replaced total cholesterol with body-mass index and predicted CVD events equally accurately. This suggested that the less costly non-laboratory absolute CVD risk score can effectively be used in resource scare settings.

The exchangeability of the non-laboratory-based score with commonly-used laboratory-based approaches has been validated in a United States population [9]. In an attempt to evaluate the effectiveness of the non-laboratory absolute CVD risk score in a developing country setting, we compared the ranking of individuals using the non-laboratory-based score with six other absolute risk scores based on laboratory testing in thirteen data sets of cross-sectional CVD risk factor surveys conducted in South Africa between 1987 and 2009.

## Methods

The main analyses in this study were based on the comparison of (10-year) individual-level absolute CVD (or coronary heart disease (CHD)) risk predictions using the non-laboratory-based [8] (Table 1) and six laboratory-based risk scores [5,10-12]. The study populations that had been used to develop each absolute risk score, the inputs required for each score, and the composite outcome that each score was designed to predict are described in Table 1. Beta-coefficients and specific details for each score are described in further detail in Additional file 1. Among the laboratory-based risk scores, the Framingham CVD risk equations are the most well-known (globally) and commonly-used in South Africa [13]. We, therefore, focused on the risk characterization comparisons between the recently developed Framingham (2008) CVD score [11] and the non-laboratory score in our study, although comparisons with the non-laboratory score were performed for all six of the laboratory-based scores.

**Table 1:** Study populations, inputs, and outcomes used to construct each of the risk scores used in this study

**Score**	**Population**^**a**^	**Inputs**	**Outcome**
Non-laboratory-based (Gaziano *et al*., 2008) [8]	NHANES I (US, 1971 to 1975)	Age, sex, smoking, diabetes, systolic blood pressure, treatment for hypertension, BMI	CVD death, MI, stroke, CHF, coronary bypass, PTCA
Framingham CVD 2008 (D’Agostino *et al*., 2008) [11]	Framingham, MA, US (1968 to 1987)	Age, sex, smoking, diabetes, systolic blood pressure, treatment for hypertension, total cholesterol, HDL cholesterol	MI, angina, coronary insufficiency, CHD death, stroke, TIA, CHF, PVD, CVD death
Framingham CVD (Anderson *et al*., 1991) [10]	Framingham, MA, (US., 1968 to 1975)	Age, sex, smoking, diabetes, systolic blood pressure, total cholesterol, HDL cholesterol	Same as above
Framingham CHD (Anderson *et al*., 1991) [10]	Same as above	Same as above	MI, CHD death, angina, coronary insufficiency
SCORE, high risk (Conroy *et al*., 2003) [4]	High risk European countries^b^	Age, sex, smoking, systolic blood pressure, total cholesterol	Death from: hypertensive disease, IHD, cerebrovascular disease
SCORE, low risk (Conroy *et al*., 2003) [4]	Low risk European countries^c^	Same as above	Same as above
CUORE (Giampaoli *et al*., 2007) [12]	Italy (1983 to 1997)	Age, sex, smoking, diabetes, systolic blood pressure, treatment for hypertension, total cholesterol, HDL cholesterol	Fatal and non-fatal MI or stroke

^a^Years indicate when baseline values were collected; ^b^applicable for all non-low risk European countries; ^c^applicable for Belgium, France, Greece, Italy, Luxembourg, Portugal, Spain, and Switzerland. BMI, body-mass index; *CHD* coronary heart disease, *CHF* congestive heart failure, *CVD* cardiovascular disease, *HDL* high-density lipoprotein, *IHD* ischemic heart disease, *MI* myocardial infarction, *NHANES* National Health and Nutrition Examination Survey, *PTCA* percutaneous transluminal coronary angioplasty, *PVD* peripheral vascular disease, *TIA* transient ischemic attack.

The South African study populations used for the comparison comprised thirteen data sets of cross-sectional CVD risk factor surveys in random samples of participants 25 to 74 years old from specific language/geography-based populations [14-20]. Data from the PURE, CRIBSA, and Phoenix studies have not been published but were provided to us from the principal investigator of each study. The data were collected using similar standardized methods and funds for the laboratory analysis were included in the research grants of each study. These studies were selected, in part, so we could assess potential differences across urban/rural status, population group, and time. Table 2 outlines the study populations, years of data collection, and basic demographics (age range and percent female) for each dataset. Because the average clinical characteristics of the Phoenix dataset have not yet been disseminated, this information is not reported in this study. The population of interest for this analysis was comprised of the aggregate collection of 14,772 individuals from all of the thirteen South African datasets.

**Table 2:** Description of the 13 cross-sectional cardiovascular disease (CVD) risk factor studies

**Dataset**	**Number**^**a**^	**Population description**	**Year(s) of data collection**	**% Female**	**Age range (years)**
DHS [22]	9,379	Representative South African	1998	60.2%	25 to 74
Aggregate	14,772	Aggregate of 13 datasets below	1987 to 2009	61.0%	25 to 74
BRISK [17]	644	African, urban Xhosa speaking	1990	55.4%	25 to 64
CRIBSA	1,003	African, urban, Xhosa speaking,	2008	64.3%	25 to 74
Mangaung [14]	718	African, urban, Sesotho speaking	1990	61.5%	25 to 74
QwaQwa [14]	782	African, rural, Sesotho speaking	1990	68.6%	25 to 74
AHA-FS urban [20]	385	African, urban Sesotho speaking	2009	76.8%	26 to 64
AHA-FS rural [20]	512	African, rural, Sesotho speaking	2007	70.1%	26 to 65
PURE, Urban	903	African, urban, Tswana-speaking	2005	58.9%	29 to 74
PURE, Rural	933	African, rural, Tswana-speaking	2005	65.2%	32 to 74
KwaZulu-Natal [15]	742	African, rural, Zulu-speaking	1999	82.5%	25 to 74
CRISIC [18]	775	Colored, urban, community	1990	50.7%	25 to 64
Mamre [19]	695	Colored, rural, community	1996	57.3%	25 to 74
KORIS [16]	5,608	White, rural, community	1987	54.1%	25 to 68
Phoenix	1,072	Indian, urban, community	2007-2008	73.6%	25 to 68

^a^random sample.

Correlation in risk characterization was based on comparisons of individual-level, score-specific rankings of absolute CVD risk for the non-laboratory-based score relative to the six laboratory-based scores. The first step in our analysis was to calculate individual-level risk predictions for each of the seven scores included in the study. Individuals were subsequently assigned ranks for each risk score by sex. These ranks were used to assess correlation in dichotomous risk characterization for the non-laboratory-based score compared to each laboratory-based risk score. Using a threshold of >20% 10-year Framingham (2008) CVD risk, individuals were characterized as ‘high’ or ‘low’ risk for each score based on their score-specific rank. We then found the risk level that was equivalent to the same proportion of events using each score. For example, if the CVD risk score using the Framingham risk score identified 15% of the population above 20% 10-year risk then we used the equivalent risk score of the non-laboratory risk score to identify 15% of the population. The CUORE risk score identified similar proportions of the population as did the Framingham CVD risk and the SCORE risk score threshold of >5% fatal events is equivalent to the Framingham CVD score. An alternative threshold of 10-year Framingham CHD risk >20% was also assessed. Percent agreement between the non-laboratory-based score and each of the laboratory-based scores was calculated by adding the proportions of individuals who were equivalently characterized as ‘high’ or ‘low’ risk by both scores. Spearman rank correlation coefficients were computed for the non-laboratory-based score compared to each of the laboratory-based scores to assess the correlations in the rankings across the full spectrum of risk thresholds. Pearson correlation coefficients were not reported because CVD risk prediction values were not normally distributed [21].

We also analyzed data from the 1998 South African Health and Demographic Survey (DHS), which has all the necessary information needed to complete the non-laboratory-based risk score [22]. This nationally representative sample allows us to partially assess whether our results from the cohorts might be generalizable for national primary CVD screening practice. The analyses involving comparison to the laboratory-based scores, however, could not be performed on the DHS data because it does not contain the blood cholesterol values needed to compute the laboratory-based risk assessments.

As a separate analysis of interest, we computed individual-level 10-year risk of CVD death based on the non-laboratory-based risk factors to assess the distribution of absolute CVD risk in the study cohorts and DHS populations. In this second analysis where we look at the 10-year risk of the DHS group we apply the CVD death only score because given the lack of a registry or widely available electronic hospital records for non-fatal MI or stroke in South Africa the only outcome that we will be able to validate in the near future is CVD death and, thus, use this score for projections. We also chose CVD death risk for these analyses to focus on the most severe events (that is, CVD death), as opposed to softer outcomes (such as angina events or revascularization procedures) that are harder to confirm currently in South Africa with limited registry data. These analyses served two functions: 1) to compare the distribution of risk in the representative DHS population to the study population; and 2) to characterize the predicted 10-year CVD mortality risk burden among South African adults.

Each of the studies in South Africa underwent independent study reviews under the local institutional review board (IRB)/ethics committees including the University of the Orange Free State Ethics Committee for Clinical Trials, the University of Free State Ethics Committee of the Faculty of Health Sciences, the Ethics Committee of the North-West University, the Ethics Committee at the University of Cape Town, the University of KwaZulu-Natal Biomedical Research Ethics Committee, and the Ethics Committee of the Medical Research Council of South Africa. Informed consent was obtained from all study participants under local IRB supervision. Only non-identifiable data sets were used and the Ethics Committee of the University of Cape Town approved of the study to evaluate CVD risk.

## Results

Table 3 shows the population characteristics of thirteen South African populations included in the main study population, the aggregate population that comprised these thirteen datasets, and the representative 1998 South Africa DHS population. The aggregate study population had similar characteristics to the DHS population with respect to sex, age and BMI but there were observed differences regarding current smokers (43.7% in DHS compared to 33.0% in the aggregate study population), diabetes (3.9% and 6.0%, respectively), blood pressure treatment (8.8% and 15.0%, respectively) and systolic blood pressure (123.9 and 133.9 mmHg, respectively). In addition to reporting the average levels of each risk factor used to calculated each of the individual-level risk predictions, the table includes the proportions of the population with >20% 10-year CVD death risk based on the non-laboratory-based risk factors (age-adjusted for the WHO Segi reference population). The proportion of the study population at high risk (19.9%) using this threshold was similar to the DHS population (17.9%). Although the proportion of the population at ‘high-risk’ ranged from 10.4% to 26.6% across the individual study datasets, there were no observable trends in the percent of the population at ‘high-risk’ by urban/rural status, language, or year of data collection. Non-African/Non-colored populations (Koris and Phoenix) had lower CVD risk (15.4% and 14.0%) compared to the study average (19.9%).

**Table 3:** The characteristics and CVD risk factor profiles of the study population 25 to 74 years old

**Dataset**	**Number**	**Female**	**Age (years)**	**Current smokers**	**Diabetes**	**Blood pressure treatment**	**Mean systolic blood pressure (mmHg)**	**Mean HDL cholesterol (mg/dl)**	**Mean Total cholesterol (mg/dl)**	**BMI**	**10-year CVD death risk >20% Non-lab**^**a**^
DHS[22]	9,379	60.2%	44.5	43.7%	3.85%^b^	8.8%	123.9	n/a	n/a	26.6	17.9%
Aggregate	14,772	61.0%	46.5	33.0%	6.0%	15.0%	133.9	50.8	206.5	26.9	19.9%
BRISK	644	55.4%	39.9	33.0%	4.0%^b^	9.0%	120.2	54.2	166.7	27.3	10.4%
CRIBSA	1,003	64.3%	43.7	27.0%	10.0%^c^	20.0%	126.3	45.3	170.7	30.1	20.6%
Mangaung	718	61.5%	46.6	35.0%	7.0%^c^	13.0%	141.9	52.6	195.1	27.3	23.1%
QwaQwa	782	68.6%	48.1	26.0%	6.0%^c^	12.0%	141.0	46.7	184.3	27.3	19.9%
AHA-FS urban	385	76.8%	44.5	22.0%	8.0%^b^	25.0%	135.8	46.6	162.3	27.7	12.1%
AHA-FS rural	512	70.1%	47.0	41.0%	10.0%^b^	51.0%	143.5	45.6	190.2	26.8	17.5%
PURE, Urban	903	58.9%	49.7	54.0%	2.0%^b^	10.0%	136.4	58.9	195.3	24.9	24.4%
PURE, Rural	933	65.2%	47.9	51.0%	1.0%^b^	6.0%	129.3	58.6	191.8	24.0	20.2%
KwaZulu-Natal	742	82.5%	49.0	15.0%	1.0%^b^	15.0%	128.1	48.0	160.7	26.1	14.9%
CRISIC	775	50.7%	44.0	55.0%	6.0%^b^	6.0%	134.1	57.5	230.8	26.2	13.6%
Mamre	695	57.3%	43.4	52.0%	6,0%^c^	47.0%	136.5	50.8	212.9	27.0	26.6%
KORIS	5,608	54.1%	47.0	27.0%	3.0%^b^	7.0%	135.1	49.6	230.2	26.6	15.4%
Phoenix	1,072	73.6%	47.6	25.0%	22.0%^c^	39.0%	131.8	50.0	214.7	28.4	14.0%

^a^Age standardized against WHO Segi (‘world’) reference population (gender weighted for South African population); ^b^Self report; ^c^GTT. See Table 2 for references. *HDL* high density lipoprotein.

Tables 4 and 5 display the Spearman rank correlation coefficients for the non-laboratory-based risk score compared to each of the six laboratory-based scores for each of the 13 study populations and the aggregate dataset. For men (Table 4) and women (Table 5) in the aggregate study population, the Spearman correlation was >0.93 for the laboratory-based scores compared to scores that were intended to predicted CVD events (that is, all scores aside from the Framingham CHD function). For comparison purposes, a 20% Framingham CVD risk and the 20% CUORE score of fatal and non-fatal events is equivalent to the SCORE risk of 5% for fatal events only. In general, the correlation coefficients were highest for the non-laboratory-based score compared to the SCORE and CUORE functions, followed by the Framingham CVD scores, then the Framingham CHD score. The ranges of correlation coefficients across the 13 study populations for the non-laboratory-based score compared to the Framingham (2008) CVD score were 0.877 to 0.964 (in men) and 0.888 to 0.950 (in women). Similar results were seen for the other risk scores with few exceptions.

**Table 4:** Spearman rank correlation results for six laboratory-based risk scores, each compared to non-laboratory-based risk score, men

**Dataset**	**Number**	**Fr**_**CVD**_**08 **[11]	**Fr**_**CVD**_**91 **[10]	**Fr**_**CHD**_**91 **[10]	**SCORE**_**h **[4]^**a**^	**SCORE**_**l **[4]^**a**^	**CUORE**[12]
Aggregate	5,751	0.939	0.950	0.883	0.985	0.986	0.957
BRISK	287	0.964	0.969	0.931	0.988	0.989	0.970
CRIBSA	357	0.963	0.967	0.922	0.987	0.988	0.974
Mangaung	276	0.952	0.961	0.900	0.985	0.986	0.967
QwaQwa	247	0.951	0.960	0.896	0.988	0.989	0.967
AHA-FS Urban	90	0.895	0.896	0.835	0.960	0.962	0.969
AHA-FS Rural	151	0.906	0.903	0.797	0.967	0.968	0.959
PURE, Urban	364	0.877	0.893	0.751	0.974	0.978	0.925
PURE, Rural	326	0.910	0.919	0.818	0.977	0.980	0.938
KwaZulu-Natal	130	0.944	0.943	0.881	0.981	0.981	0.968
CRISIC	382	0.945	0.956	0.891	0.987	0.988	0.952
Mamre	296	0.957	0.966	0.919	0.986	0.987	0.952
KORIS	2,571	0.951	0.959	0.914	0.988	0.989	0.966
Phoenix	274	0.959	0.962	0.921	0.963	0.966	0.971

^a^Framingham 10-year CVD risk of >20% of fatal and non-fatal events is equivalent to 5% risk of fatal events for SCORE. *CHD* coronary heart disease, *CVD* cardiovascular disease, *Fr* Framingham.

**Table 5:** Spearman rank correlation results for six laboratory-based risk scores, each compared to non-laboratory-based risk score, women

**Dataset**	**Number**	**Fr**_**CVD**_**08**[11]	**Fr**_**CVD**_**91 **[10]	**Fr**_**CHD**_**91 **[10]	**SCORE**_**h**[4]^**a**^	**SCORE**_**l **[4]^**a**^	**CUORE**[12]
Aggregate	9,021	0.933	0.937	0.925	0.984	0.985	0.969
BRISK	357	0.938	0.949	0.968	0.992	0.992	0.964
CRIBSA	646	0.944	0.948	0.950	0.987	0.987	0.977
Mangaung	442	0.945	0.943	0.935	0.989	0.989	0.974
QwaQwa	535	0.943	0.951	0.933	0.991	0.992	0.981
AHA-FS Urban	295	0.918	0.912	0.904	0.978	0.978	0.964
AHA-FS Rural	361	0.910	0.903	0.884	0.967	0.969	0.956
PURE, Urban	539	0.904	0.889	0.830	0.977	0.979	0.949
PURE, Rural	607	0.888	0.884	0.844	0.975	0.977	0.930
KwaZulu-Natal	612	0.944	0.958	0.936	0.991	0.992	0.981
CRISIC	393	0.950	0.944	0.950	0.990	0.990	0.961
Mamre	399	0.949	0.944	0.953	0.989	0.989	0.952
KORIS	3,037	0.950	0.951	0.937	0.991	0.991	0.974
Phoenix	798	0.909	0.901	0.895	0.964	0.965	0.966

^a^Framingham 10-year CVD risk of >20% of fatal and non-fatal events is equivalent to 5% risk of fatal events for SCORE. *CHD* coronary heart disease, *CVD* cardiovascular disease, *Fr* Framingham.

**Figure 1:**
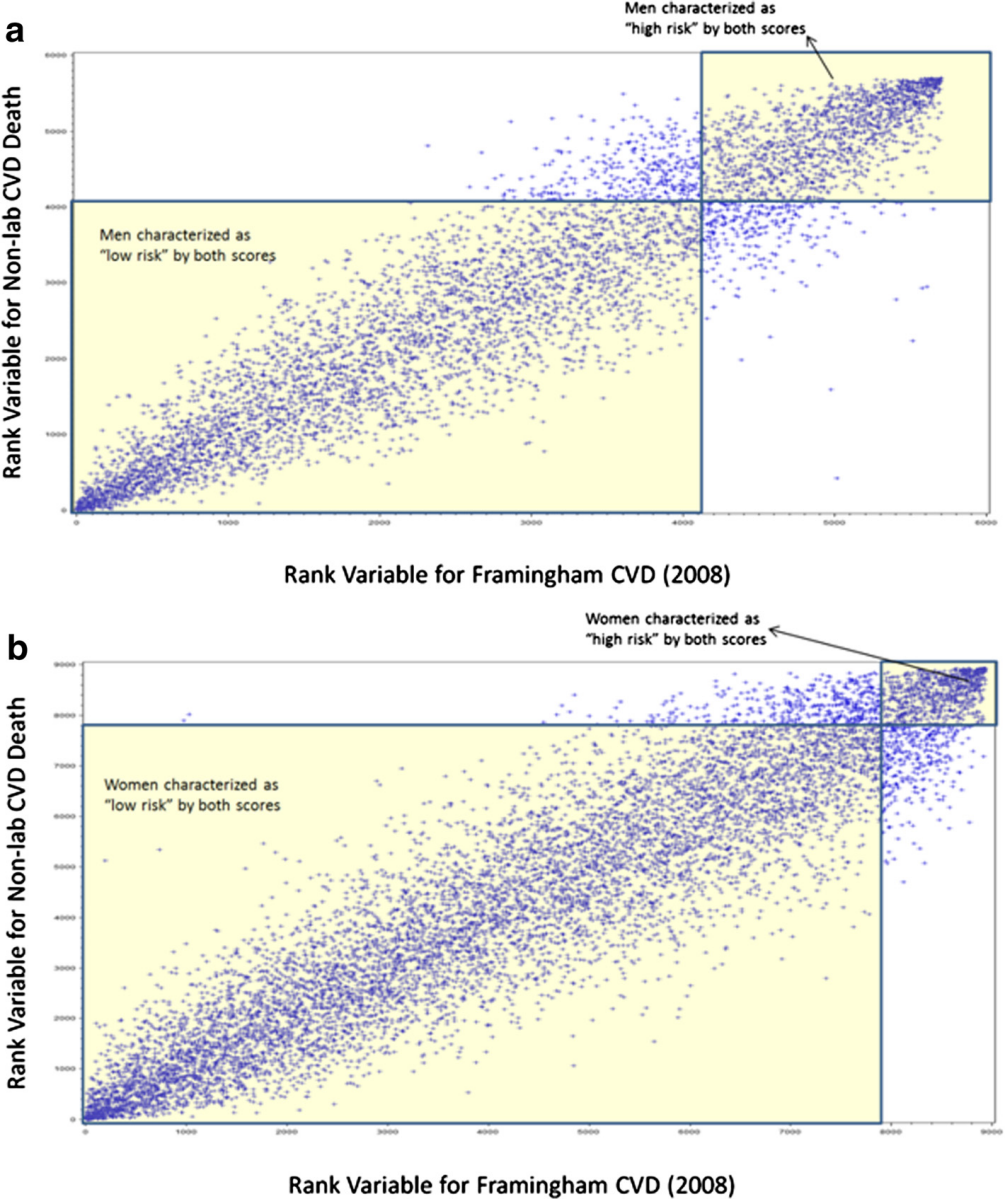
**Rank variables for the non-laboratory-based risk score are plotted against rank variables for the Framingham (2008) CVD score for adults 25 to 74 years old with complete data in the aggregate study population.** Larger ranks indicate greater CVD risk. Based on a risk threshold that corresponds to 10-year Framingham (2008) CVD risk >20%, 92.3% of men (panel **a**, shaded regions) and 94.0% of women (panel **b**, shaded regions) would be similarly characterized as high or low risk by the non-laboratory-based and Framingham (2008) CVD risk scores.

Figures 1a (for men) and 1b (for women) plot individuals in the aggregate study population based on rankings of risk (in ascending order) for the non-laboratory-based (vertical axis) and Framingham (2008) CVD (horizontal axis) risk scores. Based on a risk threshold that corresponds to 10-year Framingham (2008) CVD risk >20%, there was 89.8% and 93.9% agreement in risk characterization between the non-laboratory-based and the Framingham (2008) CVD risk scores, for men and women, respectively. Table 6 (for men) and Table 7 (for women) show the complete risk characterization agreement results for the non-laboratory-based score compared to each of the six laboratory-based scores and for each of the 13 study populations in addition to the aggregate study dataset. The risk characterization agreement findings were similar to the Spearman rank correlation results both in terms of higher agreement/correlation with CVD scores (as opposed to the Framingham CHD score).

**Table 6:** **Risk categorization results for six laboratory-based risk scores, each compared to non-laboratory-based risk score, men**^**a**^

**Dataset**	**Number**	**Fr**_**CVD**_**08**	**Fr**_**CVD**_**91**	**Fr**_**CHD**_**91**	**SCORE**_**h**	**SCORE**_**l**	**CUORE**
Aggregate	5,751	89.8%	91.0%	86.4%	95.2%	95.6%	92.5%
BRISK	287	97.9%	97.2%	97.2%	99.3%	99.3%	97.9%
CRIBSA	357	93.3%	92.2%	91.6%	93.3%	93.8%	95.0%
Mangaung	276	90.5%	91.3%	85.5%	95.6%	95.6%	93.5%
QwaQwa	247	94.3%	94.3%	91.0%	97.6%	96.7%	95.9%
AHA-FS Urban	90	88.9%	91.1%	86.7%	93.3%	95.6%	95.6%
AHA-FS Rural	151	85.6%	84.9%	78.1%	94.5%	94.5%	90.4%
PURE, Urban	364	95.7%	95.7%	92.1%	97.6%	97.6%	97.0%
PURE, Rural	326	90.1%	90.7%	88.9%	95.1%	95.1%	90.7%
KwaZulu-Natal	130	87.6%	84.5%	81.4%	93.8%	92.2%	92.2%
CRISIC	382	96.9%	96.6%	94.8%	98.7%	99.0%	97.7%
Mamre	296	94.6%	93.2%	89.8%	96.6%	96.6%	95.3%
KORIS	2,571	90.9%	91.8%	88.0%	95.6%	96.0%	93.5%
Phoenix	274	92.7%	91.2%	86.1%	90.5%	90.5%	94.9%
Aggregate^b^		92.1%	93.0%	89.6%	95.5%	95.9%	94.2%

^a^‘Agreement’ based on dichotomous risk categorization corresponding to 10-year Framingham (2008) CVD risk >20%, unless otherwise noted. For SCORE this is equivalent to a 5% fatal CVD risk; ^b^threshold of 10-year Framingham CHD risk >20%. *CHD* coronary heart disease, *CVD* cardiovascular disease, *Fr* Framingham.

**Table 7:** **Risk categorization results for six laboratory-based risk scores, each compared to non-laboratory-based risk score, women**^**a**^

**Dataset**	**Number**	**Fr**_**CVD**_**08**	**Fr**_**CVD**_**91**	**Fr**_**CHD**_**91**	**SCORE**_**h**	**SCORE**_**l**	**CUORE**
Aggregate	9,021	93.9%	94.0%	91.7%	95.8%	95.9%	96.2%
BRISK	357	97.2%	97.8%	97.8%	97.8%	97.8%	97.8%
CRIBSA	646	95.7%	95.4%	94.4%	96.6%	96.9%	97.8%
Mangaung	442	92.7%	92.3%	89.1%	95.9%	95.9%	94.5%
QwaQwa	535	92.1%	91.0%	87.7%	95.5%	95.5%	95.1%
AHA-FS Urban	295	97.3%	97.3%	96.6%	96.6%	96.6%	97.3%
AHA-FS Rural	361	92.8%	92.0%	89.7%	92.6%	92.6%	93.7%
PURE, Urban	539	92.8%	93.2%	89.0%	96.6%	97.0%	95.8%
PURE, Rural	607	94.4%	94.1%	92.1%	97.7%	98.0%	96.4%
KwaZulu-Natal	612	96.7%	97.7%	96.4%	98.2%	98.2%	99.0%
CRISIC	393	94.7%	94.9%	93.7%	97.7%	98.0%	96.5%
Mamre	399	94.0%	93.5%	91.0%	95.5%	96.0%	95.5%
KORIS	3,037	92.8%	92.8%	89.7%	95.1%	95.3%	95.0%
PHOENIX	798	91.2%	90.9%	89.2%	91.9%	92.2%	94.2%
Aggregate^b^	9,021	97.1%	97.2%	96.3%	98.1%	98.3%	97.9%

^a^‘Agreement’ based on dichotomous risk categorization corresponding to 10-year Framingham (2008) CVD risk >20%, unless otherwise noted; ^b^threshold of 10-year Framingham CHD risk >20%. *CHD* coronary heart disease, *CVD* cardiovascular disease, *Fr* Framingham.

**Figure 2:**
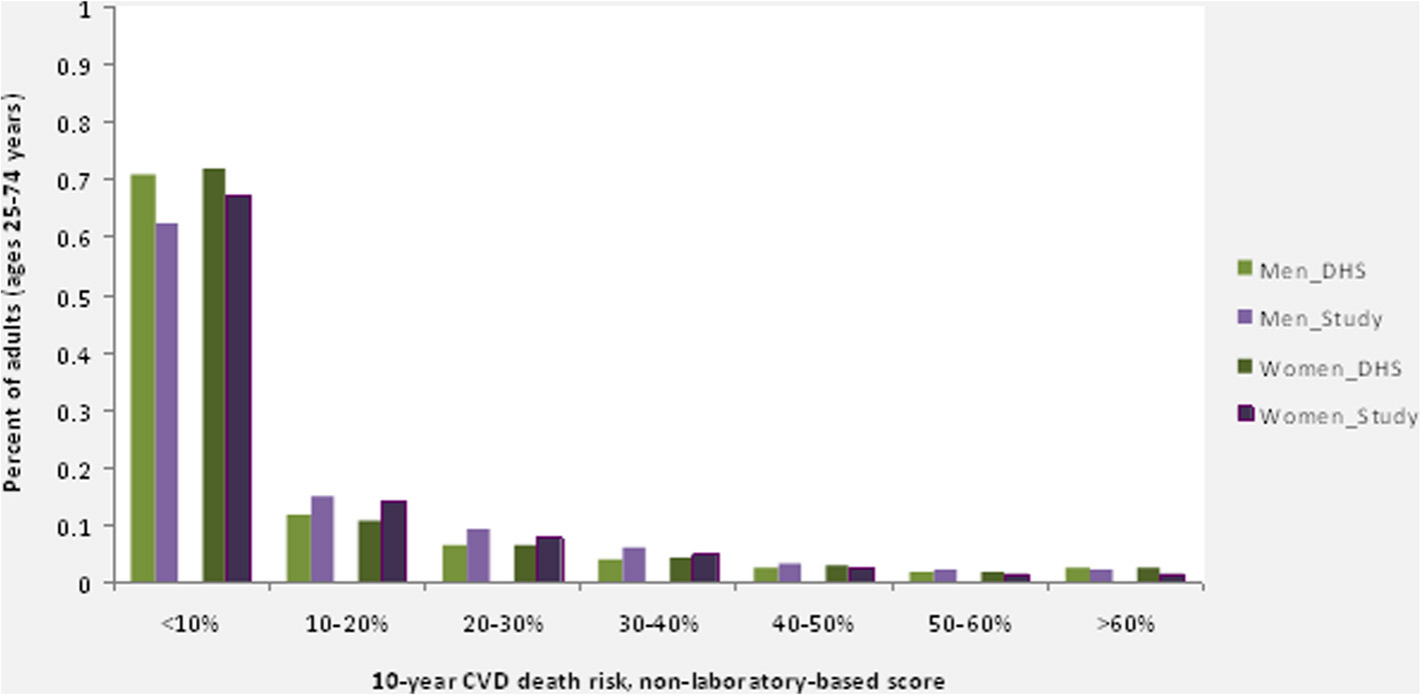
**Histograms of 10-year non-laboratory-based CVD death risk are plotted for the aggregate study population and the representative DHS (South Africa, 1998) populations by sex for adults ages 25–74 years (age-adjusted for WHO Segi ‘world’ reference population).** The study population has a slightly higher risk profile compared to the DHS population in the middle ranges (10% to 30% to 40%), although the overall distributions of risk are mostly similar between these populations for both men and women. Histogram of non-laboratory-based risk (10-year risk of CVD death) for adults in the study sample and DHS 98 population. CVD, cardiovascular disease; DHS, Health and Demographic Survey; WHO, World Health Organization.

**Figure 3:**
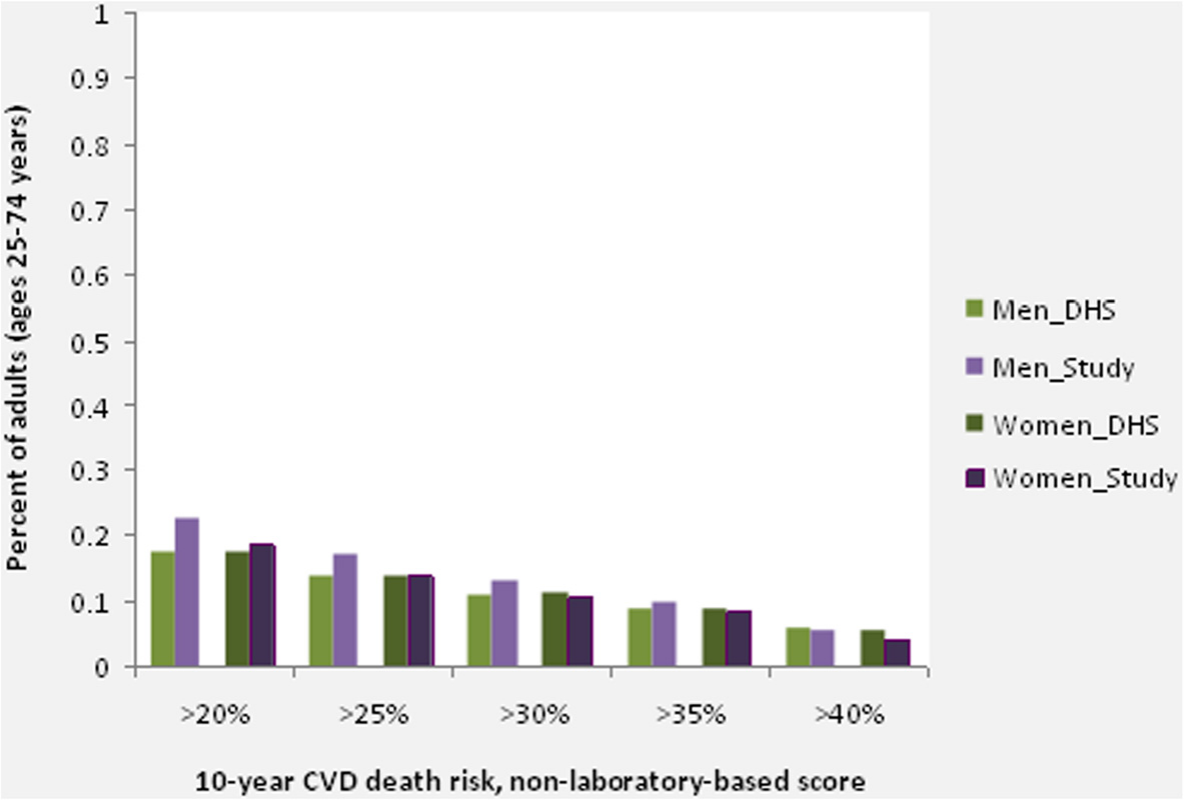
**Percentages of adults ages 25–74 years from the aggregate study population and the representative DHS (South Africa, 1998) populations that are greater than specified non-laboratory-based 10-year CVD death risk thresholds are plotted by sex (age-adjusted for WHO Segi ‘world’ reference population).** For men, the study population has a slightly higher risk profile compared to the DHS population in the lower-to-middle risk thresholds (>20% to >30%), although the overall distributions of risk are mostly similar between these populations for both men and women. Percent of adults in the study sample, and DHS 98 population, above selected non-laboratory-based thresholds (10-year CVD death risk). CVD, cardiovascular disease; DHS, Health and Demographic Survey; WHO, World Health Organization.

Figure 2 shows the distribution of 10-year non-laboratory-based CVD death risk in the aggregate study and the representative 1998 South Africa DHS populations by sex (age-adjusted to WHO Segi reference population). The largest proportion of each population belonged in the <10% risk group, although 30% of the populations had 10-year non-laboratory-based CVD death risk >10%. Approximately 2% to 3% of each population was at greater than 60% 10-year risk of dying from CVD. Figure 3 shows the percent of the same populations that were above selected 10-year CVD death risk thresholds based on non-laboratory-based risk factors. More than 17% of individuals in each population were at greater than 20% 10-year CVD death risk (which is close to the general recommended risk thresholds for treatment for medium-income countries), and 4% to 6% of the aggregate study populations were at greater than 40% 10-year CVD death risk [23]. The aggregate study population had slightly more individuals at higher risk in the lowest category considered (>20% risk), while the proportions of the aggregate study population above each threshold >25% were similar to the DHS population (the differences were less than 3.3% for each threshold, in absolute terms). There were small differences in the gender comparisons, with the aggregate study population having slightly higher proportions of men at high risk compared to the DHS population (women were similar across these populations).

## Discussion

In this study, we found that a non-laboratory-based CVD risk score, when compared to six versions of laboratory-based Framingham, SCORE and CUORE equations, similarly ranked individuals and characterized CVD risk in all 13 cohorts studied. We observed strong agreement in risk characterization between the non-laboratory-based and laboratory-based scores in all the cohorts. This was true for the aggregate of the cohorts and in each of the cohorts, which had a wide range of overall risk suggesting the non-laboratory-based risk tool performs as well in low-risk groups as it does in high-risk populations. The greatest agreement was with the SCORE risk tool with more than 96% agreement in both men and women. More than 92% of men and 97% of women were equivalently characterized as ‘high’ or ‘low’ risk by non-laboratory-based and laboratory-based scores using a risk threshold based on a threshold commonly used in guidelines (10-year CHD risk >20 [24]. At a risk of 5% in both the high and low risk populations, the SCORE equation is equivalent to the fatal and non-fatal threshold of 20% for Framingham CVD risk.

Correlation at the CVD risk threshold of >20% was highest for the laboratory-based CVD outcome scores, with the older Framingham CHD only score slightly lower. In general, the correlation was greater for women than for men. In men, using the 1991 Framingham CHD score, the correlations for two cohorts, AHA-FS rural and the PURE urban, were slightly lower (0.797, 0.751, respectively). This is important as there has been a movement for risk prediction tools to include both stroke and ischemic heart disease given that many low- and middle-income countries, including South Africa, have a higher proportion of stroke in the total CVD events. Further, reductions in most risk factors for ischemic heart disease also reduce stroke and, thus, a risk prediction tool including both is more in line with clinical activities. However, this level of correlation is still considered ‘high correlation’ being greater than 0.7 if not ‘very high correlation’ for correlations greater than 0.9 [25].

The ability to assess risk without the requirement of expensive laboratory blood tests is important in many low-income settings for multiple reasons. The first includes its ability to correctly classify patients at the thresholds that most prevention guidelines choose for initiating treatment. Other considerations include practicality, cost and feasibility, in particular providing clinicians with the opportunity to make a treatment decision during a single clinic visit. This obviates the need for a laboratory test and a second patient visit to review the results and plan management with a resultant reduction of costs and the potential for non-attendance at the follow up visit. The cost of cholesterol testing and the follow-up clinic visit in South Africa is more than US $30. Testing for cholesterol for the adult population even at a frequency of every five years would cost $110 million annually. The annual cost of treating a patient on generic simvastatin can be less than $20 [26] (less than the cost of testing and follow-up visits) which on an annual basis for all those above 20% 10-year risk would equal nearly $60 million. Thus, money saved from excessive laboratory testing can easily offset the cost of treating those at high risk with an additional savings of nearly $50 million. In addition to these savings would be the added benefit of preventing more premature deaths and disabling myocardial infarctions and strokes. Furthermore, patients, their families or members of the public, with access to a blood pressure device, can calculate their own risk and present themselves to facilities for further evaluation. This opens up the potential for increased opportunistic screening. Furthermore, it could lead to improved risk stratification for those who may be treated so that limited medications go to those who need it most.

Using the non-laboratory-based screening tool, nearly 18% of the DHS and aggregate study populations would have a 10-year CVD death risk greater than 20%. What is striking is that using the 1998 Demographic Health Survey population, the risk for women is only slightly lower than for men, which is not the case for most developed populations where men have a considerably higher risk, at least in the middle-aged populations. However, in the DHS and in many of the cohorts included in the analysis, while smoking rates tend to be higher for men, the opposite is true for diabetes and obesity and hypertension in the elderly. The obesity rates are almost double and the diabetes rate is nearly 50% greater for women than for men [27]. These differences suggest that South African women, in particular, could be facing a greater burden compared to men, especially as smoking rates for men continue to decline.

One limitation of our analysis is that the risk discrimination and validation performance of the non-laboratory-based risk score has not been validated in a longitudinal South African cohort study. The lack of reliable death data for these cross-sectional cohorts makes it impossible at this time to validate any prediction score for CVD death. Having a cohort study with death data would also allow us to assess the level of correct classification and misclassification between the different risk scores. It is also possible that the non-laboratory-based risk score may over- or underestimate the true risk, but that is true for all the risk scores compared in the analysis. This is also true for most individual risk factors on which developing countries base their guidelines. Unfortunately, few low-income countries have cohort data to make these estimates. No risk score has been validated in South Africa nor, for that matter, in most populations in low- and middle-income countries, except China [28]. However, studies to validate the risk score are ongoing in South Africa but this data will not be available for nearly five years. However, the clinical need to risk stratify patients exists now. Our results suggest that using the non-laboratory-based scores will classify people similarly in the meantime as those which rely on laboratory-based information—neither of which have been validated in developing countries. Previous analysis in the US has shown that less than 7% disagreement occurs in risk classification [9]. Further, the WHO/International Society of Hypertension (ISH) risk charts have not been validated in individual country populations but are being promoted for screening. However, all the scores we evaluated will identify the same high risk people. So, if a country is using one of the laboratory-based risk tools, it could substitute a non-laboratory based tool for it and identity the same group for less cost. The WHO/ISH risk charts were not included in our analyses because the underlying risk functions are not publicly available [23].

## Conclusions

In summary, we found that a non-laboratory-based CVD risk assessment tool ranked individuals nearly identically compared to risk scores that require additional expensive cholesterol tests. Using the non-laboratory-based risk assessment tool, we estimate that nearly 20% of the South African adult population is at high CVD risk. Health care providers that have limited resources and time in overcrowded primary health centers in South Africa can assess risk and make treatment decisions in a much more timely and inexpensive manner using results similar to those obtained using blood-based risk tools. Further, this risk factor information can be obtained non-invasively in about 5 to 10 minutes. This initial screening without blood testing could lead to the quick initiation of treatment without the added cost or inconvenience of laboratory testing. This would also minimize any potential loss to follow-up due to the extra step in testing.

## Abbreviations

BMI: Body mass index; CHD: Coronary heart disease; CHF: Coronary heart failure; CVD: Cardiovascular disease; HDL: High-density lipoprotein; IHD: Ischemic heart disease; ISH: International society of hypertension; MI: Myocardial infarction; NHANES: National Health and Nutrition Examination Survey; PTCA: Percutaneous transluminal coronary angioplasty; PVD: Peripheral vascular disease; TIA: Transient ischemic attack; WHO: World Health Organization.

## Competing interests

The authors declare that they have no competing interests.

## Authors’ contributions

TAG, AP and KS conceived of the analysis and made substantial contributions to the design of the study. KS, NL WM, GJ, CMW, AAM, AK, AS, DPN, DRP and RL made significant contributions in the acquisition of the data and interpretation of the data from their respective cross-sectional studies and the overall study. TAG and AP performed the analysis. TAG, AP and KS drafted the initial manuscript. All other authors contributed to critical revisions of the drafts and made important comments for intellectual content. All authors read and approved the final manuscript.

## Pre-publication history

The pre-publication history for this paper can be accessed here:

http://www.biomedcentral.com/1741-7015/11/170/prepub

## Supplementary Material

Additional file 1 Risk scores calculated for adults South African study population. Click here for file
