# Supplementary material for: Comparative assessment of absolute cardiovascular disease risk characterization from non-laboratory-based risk assessment in South African populations
Source: BMC Med. 2013 Jul 24;11:170. doi: 10.1186/1741-7015-11-170 (PMC3734109; doi:10.1186/1741-7015-11-170)
Supplement: Additional file 1 — Risk scores calculated for adults South African study population. [file 1741-7015-11-170-S1.doc]

| **Additional File 1**. Risk scores calculated for adults South African study population   | Variable (and beta coefficients)* | Framingham CVD 2008 (D'Agostino et al., 2008) | Framingham CHD 1991 (Anderson et al., 1991) | SCORE  (Conroy et al., 2003) | CUORE  (Giampaoli et al., 2007) | Non-laboratory-based (Gaziano et al., 2008) | | --- | --- | --- | --- | --- | --- | | Population (years for baseline values, age ranges) | Framingham, MA, U.S. (1968-1987, 30-74 years) | Framingham, MA, U.S. (1968-1975, 30-74 years) | High risk** and low risk*** European countries** (1972-88, 24-80 years) | Italy (12 random samples) (1983-1997, 35-69 years) | NHANES I, representative U.S. (1971-75, 25-74 years) | | Age | 3.061, 2.329 | several forms**** | Included***** | 0.076, 0.079 | 5.228, 6.035 | | Sex | sex-specific predictions | 18.814 | Included***** | sex-specific predictions | sex-specific predictions | | Smoking | 0.655, 0.529 | -0.390 | Included***** | 0.508, 0.773 | 0.658, 0.724 | | History of diabetes****** | 0.574, 0.692 | -0.304, -0.473 | Not included | 0.462, 0.339 | 0.511, 0.488 | | SBP | 1.933, 2.762 | -1.403 | Included***** | 0.013, 0.016 | 2.588, 2.080 | | Total cholesterol | 1.124, 1.209 | total/HDL ratio: -0.539 | Included***** | 0.006, 0.003 | Not included | | HDL cholesterol | -0.933, -0.708 | total/HDL ratio: -0.539 | Not included | -0.013, -0.015 | Not included | | Treatment of hypertension | changes coefficients for SBP to: 1.999, 2.823 | Not included | Not included | 0.490, 0.590 | 0.190, 0.257 | | BMI | Not included | Not included | Not included | Not included | 0.901, 0.872 | | Outcome | MI, angina, coronary insufficiency, CHD death, stroke, TIA, CHF, PVD, CVD death | Same as Framingham CVD 2008 | Death from: hypertensive disease, IHD, cerebrovascular disease | Death from: IHD, diabetes, cerebrovascular disease, sudden death, unknown causes. Non-fatal MI, other IHD, stroke | Death from: MI, CHF, cardiac arrest, other IHD, and cerebrovascular disease | | *Beta coefficient listed (for men, women), if included in risk score inputs, and refer to natural logs for continuous variables for Framingham CVD 2008, Framingham CHD 1991, and non-laboratory-based risk scores | | | | | | | **Applicable for all non-low risk European countries | | | | | | | ***Applicable for Belgium, France, Greece, Italy, Luxembourg, Portugal, Spain, and Switzerland | | | | | | | ****coefficient for (log(age)): -1.215, coefficient for (log (age^2^)): -1.844, coefficient for (log(age^2^)*female): 0.367 | | | | | | | *****specific risk factor coefficients not displayed due to complex equation | | | | | | | Abbreviations: National Health and Nutrition Examination Survey (NHANES), high-density lipoprotein (HDL), body-mass index (BMI), myocardial infarction (MI), coronary heart disease (CHD), transient ischemic attack (TIA), congestive heart failure (CHF), peripheral vascular disease (PVD), cardiovascular disease (CVD), ischemic heart disease (IHD), percutaneous transluminal coronary angioplasty (PTCA), systolic blood pressure (SBP) | | | | | | | | |
| --- | --- | --- | --- | --- | --- | --- | --- | --- | --- | --- | --- | --- | --- | --- | --- | --- | --- | --- | --- | --- | --- | --- | --- | --- | --- | --- | --- | --- | --- | --- | --- | --- | --- | --- | --- | --- | --- | --- | --- | --- | --- | --- | --- | --- | --- | --- | --- | --- | --- | --- | --- | --- | --- | --- | --- | --- | --- | --- | --- | --- | --- | --- | --- | --- | --- | --- | --- | --- | --- | --- | --- | --- | --- | --- | --- | --- | --- | --- | --- | --- | --- | --- | --- | --- | --- | --- | --- | --- | --- | --- | --- | --- | --- | --- | --- | --- | --- | --- | --- | --- | --- | --- | --- | --- | --- | --- | --- | --- |

**References**:

Anderson KM, Odell PM, Wilson PW, Kannel WB: **Cardiovascular disease risk profiles**. *Am Heart J* 1991, **121**(1 Pt 2):293-298.

D'Agostino RB, Sr., Vasan RS, Pencina MJ, Wolf PA, Cobain M, Massaro JM, Kannel WB: **General Cardiovascular Risk Profile for Use in Primary Care: The Framingham Heart Study**. *Circulation* 2008, **117**(6):743-753.

Conroy RM, Pyorala K, Fitzgerald AP, Sans S, Menotti A, De Backer G, De Bacquer D, Ducimetiere P, Jousilahti P, Keil U *et al*: **Estimation of ten-year risk of fatal cardiovascular disease in Europe: the SCORE project**. *Eur Heart J* 2003, **24**(11):987-1003.

Gaziano TA, Young CR, Fitzmaurice G, Atwood S, Gaziano JM: **Laboratory-based versus non-laboratory-based method for assessment of cardiovascular disease risk: the NHANES I Follow-up Study cohort**. *The Lancet* 2008, **371**(9616):923-931.

Giampaoli S, Palmieri L, Donfrancesco C, Panico S, Vanuzzo D, Pilotto L, Ferrario M, Cesana G, Mattiello A: **Cardiovascular risk assessment in Italy: the CUORE project risk score and risk chart**. *Italian Journal of Public Health* 2007, **5**(4):102-109.
